# Tunable Emission Properties of Sb^3+^/Pb^2+^ Co-Doped Cs_7_Cd_3_Br_13_ for Optical Anti-Counterfeiting Application

**DOI:** 10.3390/nano15161238

**Published:** 2025-08-13

**Authors:** Bingbing Zheng, Shuaigang Ge, Lingli Chen, Yijia Wen, Kaihuang Huang, Bingsuo Zou

**Affiliations:** 1 School of Physical Science and Technology, Guangxi University, Nanning 530004, China; 2207301190@st.gxu.edu.cn (B.Z.); 2207401036@st.gxu.edu.cn (S.G.); 2207301006@st.gxu.edu.cn (L.C.); 2207301143@st.gxu.edu.cn (Y.W.); 2207301034@st.gxu.edu.cn (K.H.); 2 State Key Laboratory of Featured Metal Materials and Life-Cycle Safety for Composite Structures, MOE Key Laboratory of New Processing Technology for Nonferrous Metals and Materials, and School of Resources, Environment and Materials, Guangxi University, Nanning 530004, China

**Keywords:** inorganic metal halides, ion doping, photoluminescence, energy transfer, anti-counterfeiting

## Abstract

Cd-based perovskite materials have the advantages of high emission efficiency and tunable emission, as well as broad application prospects in the field of optoelectronics. However, achieving multimode dynamic luminescence under UV light excitation in a single system is a great challenge. Here, we successfully prepared Sb^3+^/Pb^2+^ co-doped Cs_7_Cd_3_Br_13_ crystals by a simple hydrothermal method. Tunable emission from orange to white and then to blue, covering the wavelength range between 370 and 800 nm, was achieved by varying the doping concentration of Pb^2+^ ions in Cs_7_Cd_3_Br_13_:0.5%Sb^3+^. Temperature-dependent photoluminescence (PL) spectra and density functional theory (DFT) calculations confirm that the wide-band white-light emission of Cs_7_Cd_3_Br_13_: Sb^3+^/Pb^2+^ crystal comes from the first self-trapped exciton (STE1) of undoped Cs_7_Cd_3_Br_13_ intrinsic capture state and the emission of free excitons (FEs) and STE2 induced by the confining effect and the Jahn–Teller effect by Pb^2+^ incorporation, as well as the Sb triplet self-trapped exciton (STE3). More specifically, the samples with the best co-doped ratio exhibit significant excitation-wavelength-dependent luminescence characteristics and can realize the conversion of the emission color from white and blue to orange. Based on the tunable emission characteristics of three emission colors, the material has good prospects in encryption and anti-counterfeiting applications. This work provides a new strategy for the application of Cd-based halides in the field of anti-counterfeiting.

## 1. Introduction

Metal-halide perovskite itself has many excellent photoelectric properties, such as high photoluminescence efficiency, tunable emission characteristics, and easy preparation, which give it great application potential in the field of optoelectronics [1,2,3,4,5,6]. However, unfortunately, most metal-halide perovskites are lead-based perovskites, and their toxicity problems have seriously limited their application in some fields with high safety requirements. Therefore, in order to break through this bottleneck, the development of low-toxic or even non-toxic alternative materials can not only give full play to their great potential in the field of optoelectronics [7,8,9,10,11,12,13,14,15,16,17,18] but also help promote the application of metal-halide perovskite materials in a wider range of fields [19,20,21,22,23,24,25].

Although the use of Cd-based metal-halide perovskite is not a direct way to reduce toxicity, it shows excellent stability. When modified by metal ion doping, the optical properties of Cd-based metal-halide perovskites are comparable to Pb-based metal halides, which makes them a potential competitor for many optoelectronic applications [25,26,27,28,29,30]. The doping of metal ions with an ns^2^ electron configuration (e.g., Sb^3+^, Bi^3+^, and Sn^2+^) in metal-halide perovskites has been shown to be an effective method for achieving efficient luminescence and high stability. In the case of Sb^3+^, with its unique singlet and triplet excitons, it is one of the most studied and effective ions currently used to modulate PL and enhance emission intensity. Metal-halide perovskites doped with Sb^3+^ can form self-trapping excitons (STEs) due to their strong electron–phonon coupling properties, which, in turn, produce an efficient wide-emission band [31,32,33,34]. For example, Sb^3+^-doped Rb_3_Cd_2_Cl_7_ microcrystalline powders were obtained by a solvent thermal method. Its luminescence peak is 525 nm and has a large Stokes shift of 200 nm, which is derived from the STE. Its photoluminescence quantum yield (PLQY) is 57.47% [35]. This result shows that Sb^3+^ doping not only improves the emission efficiency of the material but also significantly enhances its stability. Sn^2+^-doped zero-dimensional (0D) Rb_4_CdCl_6_ was synthesized for the first time by a simple hydrothermal method with an emission wavelength of 485 nm [36]. Under UV light excitation, the sample with the optimal doping ratio shows a near-unity quantum efficiency (PLQY ≈ 98.04%). The large Stokes shift and high PLQY successfully elucidate that Sn^2+^ dopants play an integral role in improving the PLQY of 0D Cd-based perovskites. Gao et al. reported the Sb^3+^-doped (NH_4_)_4_CdCl_6_ halide with high-efficiency emission and stability. Under UV light excitation, the PLQY of the original (NH_4_)_4_CdCl_6_ is extremely low. However, with the introduction of Sb^3+^, the doped (NH_4_)_4_CdCl_6_ had an emission center of 570 nm and a PLQY of up to 62% [37].

In addition to Cd-based metal-halide perovskites with excellent properties achieved by doping ions, phase-selective synthesis of Cd-based halides can also be obtained by changing the synthesis method or modifying the doping ratio, such as Rb_3_Cd_2_Cl_7_ and Cs_7_Cd_3_Br_13_ crystals [38,39]. Among them, Cs_7_Cd_3_Br_13_ is a Cd-based perovskite with a special structure. Its remarkable feature is that there are two coordination structures in the structure—a single cadmium tetrahedral structure and an angle-sharing cadmium octahedral structure. This unique structure provides an ideal material for studying the effect of structure on optical properties, and enables the occupation of different doping sites and the physical separation of the luminescence center, which has not been explored in previous studies. In past studies, doping modification of Cs_7_Cd_3_Br_13_ perovskite materials is available, but most of them focus on single-doping systems. For example, Yang et al. took Cs_7_Cd_3_Br_13_ as a dopant substrate and changed the emission properties of the material by replacing Cd^2+^ with an appropriate amount of Mn^2+^ [40]. The emission of the undoped material is mainly derived from the STE emission peak at 610 nm, whereas after Mn^2+^ doping, the emission is mainly based on the ^4^T_1_→^6^A_1_ transition of Mn^2+^ ions, which combines with the intrinsic STE to significantly enhance the emission intensity. Furthermore, Chang et al. synthesized Cs_7_Cd_3_Br_13_ by the solvothermal method and increased its PLQY from 8.28% to 57.62% with appropriate Sb^3+^ doping [25]. A series of Cd-based perovskite derivatives of different crystal structures with emission wavelengths adjustable from cyan to orange (517–625 nm) were prepared by modification of Sb^3+^-doped Cs_7_Cd_3_Br_13_ via halogen substitution. Currently, these efforts typically only show a single steady-state fluorescence emission in the visible region, which significantly limits their practical application. Therefore, the development of a new type of Cd-based halide perovskite that can produce a richer luminous color switch at different excitation wavelengths will improve the security of optical anti-counterfeiting.

In this work, we successfully synthesized an all-inorganic halide perovskite with tunable emission, Sb^3+^/Pb^2+^ co-doped Cs_7_Cd_3_Br_13_. Through spectral characterization and DFT calculations, we have explored the optical properties and physical mechanisms behind it. The PL decay spectra and the temperature-dependent PL spectra reveal that the FE and STE emission of Pb^2+^ can induce a triplet state emission of Sb^3+^ by energy transfer to the STE (^3^P_1_→^1^S_0_ transition) of Sb^3+^. The DFT calculations indicate that the introduction of Sb^3+^ and Pb^2+^ creates new energy levels, leading to a narrowing of the bandgap while making the band edges more localized. By varying the doping ratio of Sb^3+^/Pb^2+^, a tuned emission from orange to white to blue is achieved. At the excitation wavelengths of 310, 340, and 365 nm, the optimal co-doping ratio can enable the emission color flexible conversion between white, blue, and orange. The dynamic excitation-wavelength-dependent emission behavior (i.e., triple-mode luminescence from a single composition under different UV excitations) provides a simple yet effective approach for anti-counterfeiting applications. Based on the multimode dynamic luminescence characteristics of Sb^3+^/Pb^2+^ co-doped Cs_7_Cd_3_Br_13_ phosphor under different excitation wavelengths, a demonstration of tertiary fluorescence anti-counterfeiting applications is presented. This work offers new concepts and approaches for the application of Cd-based halide perovskites in the field of anti-counterfeiting, which is expected to promote the development of anti-counterfeiting technology and meet the needs of modern society for high-security anti-counterfeiting materials.

## 2. Materials and Methods

### 2.1. Materials

Cesium bromide (CsBr, 99.99%), Lead bromide (PbBr_2_, 99.99%), Antimony trioxide (Sb_2_O_3_, 99.5%), and Cadmium acetate dihydrate (Cd(CH_3_COO)_2_·2H_2_O, 99.8%) were purchased from Aladdin (Shanghai, China). Hydrogen bromide (HBr, 40 wt.%) was purchased from Macklin (Shanghai, China). Anhydrous ethanol (CH_3_CH_2_OH, 99.9%) was purchased from Nanning Blue Sky Experimental Equipment Co., Ltd (Nanning, China). All these chemical agents were used without further purification.

### 2.2. Sample Synthesis

A series of crystals based on Cs_7_Cd_3_Br_13_ as the host lattice were synthesized via a hydrothermal method by combining stoichiometric amounts of the starting materials. For a typical synthesis of undoped Cs_7_Cd_3_Br_13_, 2.33 mmol CsBr and 1.00 mmol Cd(CH_3_COO)_2_·2H_2_O were mixed with 3 mL of HBr in a 25 mL Teflon liner. The mixture was heated to 180 °C for 12 h and then cooled naturally to room temperature. The precipitated crystals were washed three times with ethanol and dried in an oven at 60 °C for 12 h. The synthesis of Cs_7_Cd_3_Br_13_:x%Sb^3+^ is similar to that of the undoped Cs_7_Cd_3_Br_13_ crystal, except that an Sb precursor solution was prepared beforehand. Specifically, 0.05 mmol of Sb_2_O_3_ was dissolved in 1 mL of HBr to prepare a 0.05 mmol/mL Sb precursor solution, and then different volumes of this solution were added according to the doping ratio. Similarly, Cs_7_Cd_3_Br_13_:y%Pb^2+^ was synthesized using the same method, with the only difference being that different amounts of PbBr_2_ were added to the reaction mixture. For the synthesis of the Cs_7_Cd_3_Br_13_:x%Sb^3+^, y%Pb^2+^ co-doped crystal, the experimental process was as follows: Firstly, prepare 0.05 mmol/mL Sb precursor solution (0.05 mmol Sb_2_O_3_ dissolved in 1 mL HBr) and 0.1 mmol/mL Pb precursor solution (0.25 mmol PbBr_2_ dissolved in 2.5 mL HBr) separately; Then, add 2.33 mmol CsBr and 1 mmol Cd(CH_3_COO)_2_·2H_2_O to a 25 mL polytetrafluoroethylene container, and inject 3 mL HBr as the solvent; Next, 100 μL of Sb precursor solution (corresponding to 0.05% Sb^3+^ doping) was fixed, and different volumes of Pb precursor solution were added according to the doping concentration ratio; Finally, seal the reaction system in a stainless steel autoclave, react at 180 °C for 12 h, then cool naturally, and the obtained product is washed three times with ethanol and dried at 60 °C for 12 h, and finally the target crystal material is obtained.

### 2.3. Preparation of Anti-Counterfeiting Pattern

A hollow template with a “potted” pattern was used. The approximate dimensions of each component are as follows: the area of the “flower” shape measures about 1.4 cm × 1.5 cm, the area of the “leaf” shape is approximately 1.6 cm × 0.8 cm, and the area of the “pot” shape is approximately 1.3 cm × 1.4 cm. Firstly, the samples of Cs_7_Cd_3_Br_13_:0.5%Sb^3+^, Cs_7_Cd_3_Br_13_:5%Pb^2+^, and Cs_7_Cd_3_Br_13_:0.5%Sb^3+^, 4%Pb^2+^ were each placed into a mortar and ground three times to obtain fine powders. Subsequently, the hollow template was placed on a black cardboard base. Finally, the mixture of Cs_7_Cd_3_Br_13_:0.5%Sb^3+^ powder and epoxy resin was filled into the “pot,” the mixture of Cs_7_Cd_3_Br_13_:5%Pb^2+^ powder and epoxy resin was filled into the “leaf,” and the mixture of Cs_7_Cd_3_Br_13_:0.5%Sb^3+^, 4%Pb^2+^ powder and epoxy resin was filled into the “flower”.

## 3. Results and Discussion

**Figure 1:**
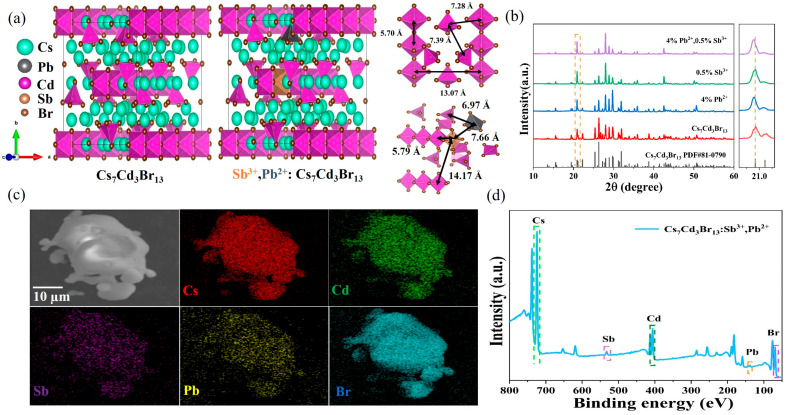
(**a**) Schematic diagram of the crystal structure of Cs_7_Cd_3_Br_13_ and Cs_7_Cd_3_Br_13_: Sb^3+^/Pb^2+^. (**b**) XRD patterns of Cs_7_Cd_3_Br_13_, Cs_7_Cd_3_Br_13_:Pb^2+^, Cs_7_Cd_3_Br_13_:Sb^3+^, and Cs_7_Cd_3_Br_13_:Sb^3+^/Pb^2+^ crystals (left), with the yellow dashed rectangle delineates the 2θ range of 20.6°–21.3°. Enlarged images of XRD spectra at the 2θ range of 20.6°–21.3° (right), with the yellow dotted line denotes the position corresponding to 2θ of 20.8°. (**c**) SEM image of Cs_7_Cd_3_Br_13_:0.5%Sb^3+^, 4%Pb^2+^ single crystal and EDS mapping images of Cs, Cd, Sb, Pb, and Br elements in Cs_7_Cd_3_Br_13_:0.5%Sb^3+^, 4%Pb^2+^ crystal. (**d**) The XPS surveys of Cs_7_Cd_3_Br_13_:0.5%Sb^3+^, 4%Pb^2+^.

The Cs_7_Cd_3_Br_13_ crystal belongs to the centrosymmetric structure with the *I4/mcm* space group [41]. The left panel of Figure 1a shows the crystal structure of Cs_7_Cd_3_Br_13_, and the middle panel shows the crystal structure of Cs_7_Cd_3_Br_13_:Sb^3+^/Pb^2+^. This structure is composed of both octahedral [CdBr_6_]^4−^ and tetrahedral [CdBr_4_]^2−^. The right panel of Figure 1a shows the distance of the octahedral center and the distance of the tetrahedral center. The octahedra [CdBr_6_]^4−^ share opposite corners to form linear anion chains, with the distance between the two nearest Cd-Cd in the chain is 5.70 Å and the distance between the Cd-Cd of the two nearest chains is 13.07 Å. The tetrahedra [CdBr_4_]^2−^ are isolated by Cs ions, with the distance between the two nearest Cd-Cd is 7.39 Å. The nearest distance between the center of the octahedron and the center of the tetrahedron is 7.28 Å. Therefore, Sb^3+^/Pb^2+^ can substitute for Cd^2+^ in two ways. The tetrahedral and octahedral formation energy of Sb replacing Cd was calculated in the work of Cs_7_Cd_3_Br_13_ doping Sb^3+^. The calculation results indicate that the energy of the [CdBr_6_]^4−^ octahedron is about 0.25 eV lower than that of the tetrahedron, so the [CdBr_6_]^4−^ octahedron is more easily replaced by the [SbBr_6_]^3−^ octahedron [25]. The crystal orbital Hamilton population (COHP) of Pb^2+^/Mn^2+^ co-doped Cs_7_Cd_3_Br_13_ crystals was calculated to analyze the chemical interactions between atoms in solids. The more positive value of -COHP indicates fewer antibonding states and more bonding states. The Pb-Br bond of the octahedron has a smaller -COHP value than that of the tetrahedron, indicating that Pb^2+^ preferentially occupies the [CdBr_4_]^2−^ tetrahedral sites. [42] Therefore, in this study, Sb occupies the octahedral sites while Pb occupies the tetrahedral sites. In this configuration, the distance between the two nearest Sb-Cd in the chain is 5.79 Å, and the distance between the Sb-Cd of the two nearest chains is 14.17 Å. The distance between the two nearest Pb-Cd is 6.96 Å. The distance between the two nearest Sb-Pb is 7.66 Å. The XRD patterns of undoped Cs_7_Cd_3_Br_13_ and co-doped Cs_7_Cd_3_Br_13_ with different ratios of Sb^3+^/Pb^2+^ are shown in Figure 1b and Figure S1. Compared with the PDF card of Cs_7_Cd_3_Br_13_ (PDF#81-0790), the position of the diffraction peak does not change significantly, which indicates that the introduction of Sb^3+^ and Pb^2+^ will not change the crystal structure of Cs_7_Cd_3_Br_13_. Moreover, through further analysis of the amplified XRD pattern (20.6°–21.3°), it can be clearly seen that the addition of Sb^3+^ will make the diffraction peak shift to a high angle, and the addition of Pb^2+^ will make the diffraction peak shift to a small angle. This shift is attributed to lattice shrinkage caused by the replacement of Cd^2+^ (~95 pm) with the smaller ionic radius of Sb^3+^ (~76 pm) [43]. In addition, with the introduction of Pb^2+^, the diffraction peak at 20.8° shifts to a smaller angle, indicating the expansion of the perovskite lattice due to the replacement of Cd^2+^ (~95 pm) by Pb^2+^ (~119 pm) with a larger ionic radius [44]. The morphology and element distribution of undoped Cs_7_Cd_3_Br_13_ and Cs_7_Cd_3_Br_13_:0.5%Sb^3+^, 4%Pb^2+^ powders were characterized using scanning electron microscopy (SEM) and energy dispersive spectroscopy (EDS). The SEM image and element mapping of Cs_7_Cd_3_Br_13_ are shown in Figure S2, and the elements Cs, Cd, and Br are evenly distributed. The SEM image and element mapping of Cs_7_Cd_3_Br_13_:0.5%Sb^3+^, 4%Pb^2+^ are presented in Figure 1c. Cs, Cd, Sb, Pb, and Br elements are evenly distributed, indicating that Sb and Pb were successfully incorporated into the lattice of Cs_7_Cd_3_Br_13_. The effective doping concentrations of Sb and Pb of Cs_7_Cd_3_Br_13_:0.5%Sb^3+^, 4%Pb^2+^ were measured by the EDS, and the atomic percentage of elements was presented in Figure S3. The atomic percentage of Sb is 0.21%, and the atomic percentage of Pb is 1.09%. By controlling the elemental ratios of the corresponding raw materials, the resulting composition is in good agreement with the target product. The X-ray photoelectron spectra (XPS) of Cs_7_Cd_3_Br_13_:0.5%Sb^3+^, 4%Pb^2+^ samples are shown in Figure 1d, showing the characteristic peaks of Cs, Cd, Sb, Pb, and Br. As shown in Figure S4, Cs_7_Cd_3_Br_13_ is located at the peaks of 404.95 and 411.8 eV, belonging to Cd^2+^ 3d_5/2_ and Cd^2+^ 3d_3/2_, respectively. Located at 68.3 eV belongs to Br 3d. Compared with undoped Cs_7_Cd_3_Br_13_, Cs_7_Cd_3_Br_13_:0.5%Sb^3+^, 4%Pb^2+^ shows two new peaks at 533.2 and 531.75 eV, which are attributed to Sb 3d_3/2_ and Sb 3d_5/2_, respectively. In addition, Cs_7_Cd_3_Br_13_:0.5%Sb^3+^, 4%Pb^2+^ also showed the characteristic peaks of Pb 4f_5/2_ (143.05 eV) and Pb 4f_7/2_ (138.2 eV). Therefore, the existence of Sb^3+^ and Pb^2+^ is further confirmed in the doped sample. In the Cs_7_Cd_3_Br_13_:0.5%Sb^3+^, 4%Pb^2+^ sample, the characteristic peaks of Cd 3d and Br 3d were slightly shifted, while the binding energy of Cs 3d remained unchanged. The results show that Sb^3+^ and Pb^2+^ are most likely to occupy the position of Cd^2+^ in the lattice.

**Figure 2:**
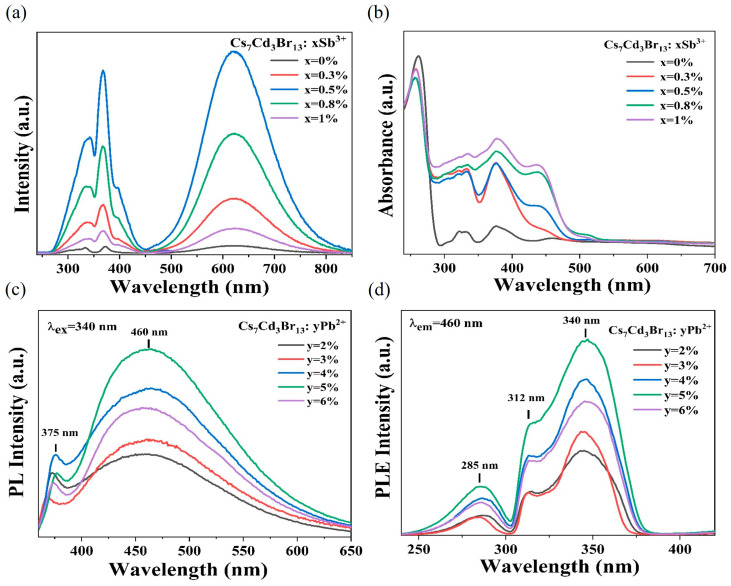
(**a**) PLE (λ_em_ = 620 nm) and PL (λ_ex_ = 370 nm) spectra of Cs_7_Cd_3_Br_13_ and Cs_7_Cd_3_Br_13_:xSb^3+^. (**b**) Optical absorption spectra of the Cs_7_Cd_3_Br_13_:xSb^3+^ sample. (**c**) PL (λ_ex_ = 340 nm) and (**d**) PLE (λ_em_ = 460 nm) spectra of Cs_7_Cd_3_Br_13_:yPb^2+^ (y = 2–6%).

The PLE and PL spectra of Cs_7_Cd_3_Br_13_ and Cs_7_Cd_3_Br_13_:x%Sb^3+^ samples are shown in Figure 2a. The undoped Cs_7_Cd_3_Br_13_ has a very weak emission band at 615 nm. Compared with the original Cs_7_Cd_3_Br_13_, the PL intensity after doping Sb^3+^ is significantly enhanced, emitting bright orange emission near 620 nm with a full-width-at-half-maximum (FWHM) of 145 nm under 370 nm excitation. Whose 620 nm band of Cs_7_Cd_3_Br_13_:x%Sb^3+^ has a Stokes shift of 250 nm, which indicates that its self-absorption is negligible, resulting in efficient emission out of the STE. With the increasing Sb^3+^ doping, the emission intensity first increases and then decreases due to the concentration quenching effect, and the emission intensity reaches its maximum when the Sb^3+^ doping concentration is 0.5%. Figure 2b displays the optical absorption spectra of undoped Cs_7_Cd_3_Br_13_ and Cs_7_Cd_3_Br_13_:x%Sb^3+^ samples. The characterization results indicate that the undoped Cs_7_Cd_3_Br_13_ shows a distinct absorption edge at 295 nm. Below this edge, there are three weak bands at 330, 370, and 460 nm that may be the absorption of the STEs for their complicated structures in this compound. This STE emission at about 615 nm and PLE can be verified by Figure 2a, which is well in agreement with the absorption spectra. The PLE spectra of the undoped Cs_7_Cd_3_Br_13_ and its doped Sb^3+^ are shown on the left of Figure 2a, which more clearly show several excitation peaks at 292, 303, 322, 332, and 375 nm, consistent with the reported literature [45]. The absorption spectra of Sb^3+^ doped samples exhibit a series of new absorption bands in the region of 280–500 nm, that of the charge transfer out of the Sb-Br-Cd octahedron. The excited states of Sb^3+^ ions (ns^2^ electronic configuration) have singlet and triplet states in the gap, where ^1^S_0_→^1^P_1_ transitions are allowed, ^1^S_0_→^3^P_1_ is partially allowed to transition, and ^1^S_0_→^3^P_2_ and ^1^S_0_→^3^P_0_ transitions are forbidden [46,47]. Below the band out of Sb in the range 450–540 nm, there are two bands possibly from the Cd states due to the Sb and Cd d orbital interactions, which give no emissions or low Cd STE emission.

The PL lifetime spectrum reveals the underlying mechanism of emission processes. The decay curves can be fitted using a biexponential function.

(1) $$I\left(t\right)=I\left(0\right)A_{1}e^{-t/\tau_{1}}+A_{2}e^{-t/\tau_{2}}$$

*I*(0) and *I*(*t*) are the emission intensities of time 0 and t, respectively, τ_1_ and τ_2_ are the two decay times of the two exponential components, and *A*_1_ and *A*_2_ are the amplitudes of the two exponential components. In addition, the average PL lifetime is calculated by the following formula:

(2) $$\tau_{\mathrm{ave}}\;=\;\frac{A_{1}\tau_{1}^{2}\;+\;A_{2}\tau_{2}^{2}}{A_{1}\tau_{1\;}+\;A_{2}\tau_{2}}$$

As shown in Figure S5a, the decay curves of Cs_7_Cd_3_Br_13_ and Cs_7_Cd_3_Br_13_:x%Sb^3+^ monitored at 620 nm can be well fitted using a biexponential function. The undoped Cs_7_Cd_3_Br_13_ is 2.80 μs, indicating that its emission is from the STE1 of Cd^2+^-Cl cluster [43]. Table S1 shows that the short-lived PL lifetime of Cs_7_Cd_3_Br_13_:0.5%Sb^3+^ sample is 2.58 μs (99.89%), whereas the long-lived PL lifetime is 18.72 μs (0.11%). These lifetimes may originate from the triplet STE emission of the Sb^3+^-Cl cluster. The short-lived PL lifetime is attributed to the ^3^P_1_→^1^S_0_ transition of the Sb^3+^-Cl cluster, and the long-lived PL lifetime may be caused by interaction with other clusters.

Figure 2c,d shows PL and PLE spectra of Cs_7_Cd_3_Br_13_:y%Pb^2+^, respectively. The PLE spectrum of Cs_7_Cd_3_Br_13_:y%Pb^2+^ consists of three bands, and its intensity varies with the concentration of Pb^2+^. The PL spectrum of Cs_7_Cd_3_Br_13_:y%Pb^2+^ shows a narrow band at 375 nm and a wide-emission band at 460 nm under 340 nm excitation [48]. When the doping concentration is 5%, the emission intensity becomes the strongest, and the PL intensity decreases after the Pb concentration increases due to the concentration quenching effect. The narrow emission band at 375 nm in Figure 2c may be caused by FE emission. The PL decay curves of Cs_7_Cd_3_Br_13_:y%Pb^2+^ are shown in Figure S5b, and the specific fitting values are shown in Table S2. The average decay lifetime of Cs_7_Cd_3_Br_13_:5%Pb^2+^ samples is 383.35 ns at 460 nm, which is similar to the wide-band emission average decay lifetime of Pb^2+^-doped Cd-based halide perovskites recently reported because of the confined exciton formation [26,49]. This suggests that the wide-emission band at 460 nm is caused by Pb^2+^ induced STE2 emission, and surrounding Pb^2+^, there are several CdBr_x_ tetrahedra.

**Figure 3:**
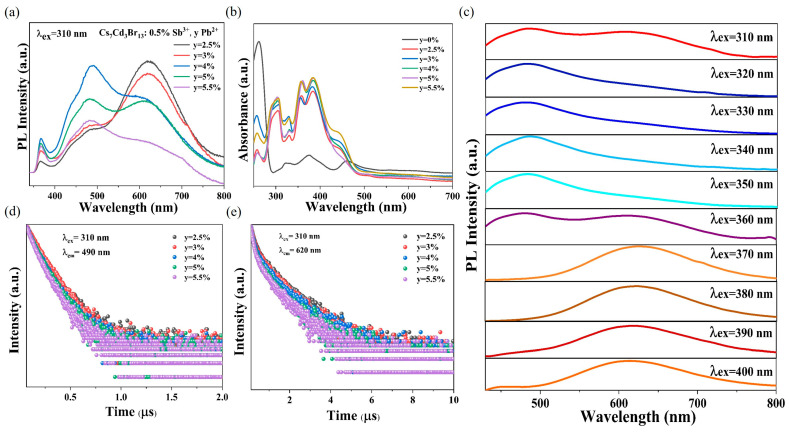
(**a**) PL spectra of Cs_7_Cd_3_Br_13_:0.5%Sb^3+^, yPb^2+^ (y = 2.5–5.5%). (**b**) Optical absorption spectra of the Cs_7_Cd_3_Br_13_:0.5%Sb^3+^, yPb^2+^ sample. (**c**) PL spectra of Cs_7_Cd_3_Br_13_:0.5%Sb^3+^, 4%Pb^2+^ samples under different excitation. (**d**) Decay curve of Cs_7_Cd_3_Br_13_:0.5%Sb^3+^, yPb^2+^ sample under 310 nm laser irradiation (monitored at 490 nm). (**e**) Decay curve of Cs_7_Cd_3_Br_13_:0.5%Sb^3+^, yPb^2+^ sample under 310 nm laser irradiation (monitored at 620 nm).

Figure 3a shows the PL spectra of Sb^3+^/Pb^2+^ co-doped Cs_7_Cd_3_Br_13_ samples at 310 nm excitation. Cs_7_Cd_3_Br_13_:0.5%Sb^3+^ exhibits the best optical properties, so the optimal Sb^3+^ doping level is chosen as 0.5%. With the introduction of Pb^2+^, the intensity of the 620 nm emission band gradually decreases. Meanwhile, a new wide-emission band appears at 490 nm, likely due to energy transfer between excitons in [PbBr_4_]^2−^ and the STE of [SbBr_6_]^3−^. When the concentration of Pb^2+^ is greater than 4%, the emission band intensity of Pb^2+^ decreases due to the concentration quenching effect. Figure S6 shows the normalized PL spectra of Cs_7_Cd_3_Br_13_:0.5%Sb^3+^, y%Pb^2+^ under 310 nm excitation. Compared with the normalized PL spectra of different doping concentrations, the emission center is roughly unchanged, indicating that the emission band should come from the same excitation source. By changing the concentration of Pb^2+^ in Cs_7_Cd_3_Br_13_:0.5%Sb^3+^, y%Pb^2+^ samples, and maintaining the concentration of Sb^3+^ unchanged, the emission color of the sample can achieve three color emission from orange, to white, and then blue with rising Pb concentration. When the concentration of Pb^2+^ changes from 2.5% to 5.5%, the CIE coordinates of PL produced by Cs_7_Cd_3_Br_13_:0.5%Sb^3+^, y%Pb^2+^ vary from (0.440,0.390) to (0.301,0.337) under 310 nm excitation (Figure S7a). Notably, the CIE coordinates of Cs_7_Cd_3_Br_13_:0.5%Sb^3+^, 4%Pb^2+^ become (0.3172,0.3484), corresponding to white-light emission (Figure S7b). Figure S8a,b shows PL spectra of Cs_7_Cd_3_Br_13_:0.5%Sb^3+^, y%Pb^2+^ under excitation of 340 nm and 370 nm, respectively. It can be seen that the PL spectrum mainly presents blue emission near 485 nm under 340 nm excitation. In addition, Cs_7_Cd_3_Br_13_:0.5%Sb^3+^, y%Pb^2+^ samples show an orange emission band again at about 620 nm under 370 nm excitation. This observation indicates that 370 nm can excite only the Sb^3+^ octahedra in this compound.

Figure 3b shows the absorption spectra of Cs_7_Cd_3_Br_13_:0.5%Sb^3+^, y%Pb^2+^ samples. Compared with the absorption spectra of only doped Sb^3+^ (Figure 2b), the introduction of Pb^2+^ results in the appearance of a new absorption band at 360 nm and an enhancement of the band intensity at 305 nm. The co-doped absorption region at 250–500 nm is formed by the superposition of the optical absorption of Sb^3+^ and Pb^2+^ doping. Figure S9a,b shows the PLE spectra of Cs_7_Cd_3_Br_13_:0.5%Sb^3+^, y%Pb^2+^ at 490 and 620 nm emission, respectively. The PLE spectra are consistent with the band profile of the PLE spectra of single-doped Pb^2+^ (Figure 2d) at 490 nm emission, indicating that the co-doped wide-band emission of 490 nm is derived from the STE2 emission of Pb^2+^. Comparing the 620 nm emission of the co-doped PLE spectrum with that of the single-doped Sb^3+^ PLE spectrum (Figure 2a), it can be found that the band of the PLE spectrum is the same, indicating that the 620 nm wide-band emission of the co-doped PL spectrum comes from the triplet state emission of Sb^3+^ or the Cd-Sb band mixing because the Sb doping can shift the absorption band edge and PLE band edge to red and enhance their intensity. Figure S10a,b shows the normalized PLE spectra of Cs_7_Cd_3_Br_13_:0.5%Sb^3+^, y%Pb^2+^ under emission of 490 and 620 nm, respectively. It can be seen from Figure S10a that the band shape of the two emission PLE is similar for 490 nm emission, independent of doping concentration. However, for the 620 nm emission, the PLE band redshifts with higher Pb concentration over Pb 5.5%. This may imply that more Pb^2+^ may occupy the tetrahedra, which shifts the band edge due to Pb-Cd interaction. Therefore, the wide band at 620 nm has some other source of excitation because Pb is involved in the interaction between Sb and Cd. Figure 3c shows the PL spectra of Cs_7_Cd_3_Br_13_:0.5%Sb^3+^, y%Pb^2+^ samples under excitation wavelengths ranging from 310 to 400 nm. The results indicate that the emission color can be tuned with the composition of three metal elements and the excitations on their individual levels, realizing the emission of white, blue, and orange light at different excitations, with the characteristics of tunable emission color. Figure S11 shows the PLQY of Cs_7_Cd_3_Br_13_:0.5%Sb^3+^, y%Pb^2+^ sample under 310 nm excitation. The sample with 4% Pb^2+^ doping its PLQY is the highest about 17.2%. This low QY value in this doped compound is most originated from the lowest energy level out of Cd ions located in the chained octahedra, which can be populated but its energy relaxation via exchange is efficient within the Cd-Cl chain, but not the doped ions, so it is hard to emit light efficiently due to the more nonradiative decay pathways. Consequently, these factors result in a relatively low PLQY for the co-doped samples.

We analyzed the PL decay curves of all samples at emission wavelengths of 490 and 620 nm, as shown in Figure 3d,e. Furthermore, the average PL lifetimes are calculated using exponential fitting, and the detailed parameters are provided in Tables S3 and S4. With the increasing Pb^2+^ concentration, the PL lifetime at 490 and 620 nm gradually decreases, indicating that energy transfer occurs as a result of the concentration quenching effect. The average PL lifetime of the Cs_7_Cd_3_Br_13_:4%Pb^2+^ sample at the emission of 460 nm is 152.91 ns. The average PL lifetime of the Cs_7_Cd_3_Br_13_:0.5%Sb^3+^, 4%Pb^2+^ sample at 490 nm decreases to 121.04 ns, indicating that the decay of the excited state is faster due to the introduction of Sb^3+^. This indicates that in the Cs_7_Cd_3_Br_13_:0.5%Sb^3+^, 4%Pb^2+^ sample, energy transfer occurs from Pb-STE_2_ to Sb-STE_3_ under 310 nm excitation. The Dexter transfer efficiency from Pb-STE_2_ to Sb-STE_3_ can be calculated using the following formula:

(3) $$\eta_{x}=1\;-\;\frac{\tau_{x}}{\tau_{0}}$$

where τ_0_ represents the average PL lifetime of Cs_7_Cd_3_Br_13_:4%Pb^2+^ sample at 310 nm excitation and 460 nm emission, τ_x_ denotes the average PL lifetime of Cs_7_Cd_3_Br_13_:0.5%Sb^3+^, 4%Pb^2+^ sample at 310 nm excitation and 490 nm emission, and *η_x_* is the Dexter transfer efficiency. It can be calculated that the Dexter transfer efficiency of Cs_7_Cd_3_Br_13_:0.5%Sb^3+^, 4%Pb^2+^ sample is approximately 21%.

**Figure 4:**
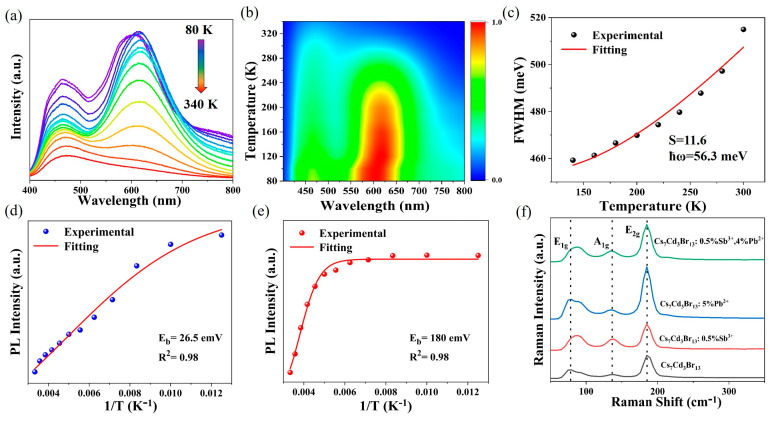
(**a**) Temperature-dependent PL spectra of Cs_7_Cd_3_Br_13_:0.5%Sb^3+^, 4%Pb^2+^ with an excitation wavelength of 310 nm. (**b**) Pseudocolor map from temperature-dependent PL spectra of Cs_7_Cd_3_Br_13_:0.5%Sb^3+^, 4%Pb^2+^. (**c**) Fitted S factor (FWHM of emission at 620 nm). (**d**) Fitting of E_b (620)_ (PL intensity@620 nm, 1/T). (**e**) Fitting of E_b (490)_ (PL intensity@490 nm, 1/T). (**f**) Raman spectra of Cs_7_Cd_3_Br_13_ and Cs_7_Cd_3_Br_13_:x%Sb^3+^, y%Pb^2+^ excited by 532 nm laser.

At low temperatures, the materials exhibit emission behavior that is not observed at room temperature. The temperature-dependent PL spectra of Cs_7_Cd_3_Br_13_ and Cs_7_Cd_3_Br_13_:5%Pb^2+^ are shown in Figure S12. The undoped Cs_7_Cd_3_Br_13_ exhibits a double band emission at low temperatures, corresponding to the high-energy (HE) band originating from the [CdBr_4_]^2−^ tetrahedron and the low-energy (LE) band associated with the [CdBr_6_]^4−^ octahedron. At low temperatures, the vibration of the tetrahedron is weakened, and nonradiative dissipation is reduced, resulting in luminescence at 500 nm. However, as the temperature increases, the vibration of the [CdBr_4_]^2−^ tetrahedron is enhanced, leading to the disappearance of the emission band at 500 nm. Meanwhile, the [CdBr_6_]^4−^ octahedron produces an emission band at 610 nm at low temperatures. As the temperature rises, the enhancement of lattice vibrations and structural distortions increases nonradiative dissipation, causing a slight redshift of the emission band, a decrease in emission intensity, and an increase in the FWHM [25,39,46]. When the temperature is below 200 K, the STE band of Cs_7_Cd_3_Br_13_:5%Pb^2+^ blueshifts with the increase in temperature, indicating that thermal expansion dominates the bandgap behavior. At higher temperatures, a slightly redshifted STE band is observed, attributed to the non-negligible electron–phonon coupling effect [50]. Figure 4a shows the temperature-dependent PL spectra of Cs_7_Cd_3_Br_13_:0.5%Sb^3+^, 4%Pb^2+^ at 310 nm excitation. Figure 4b shows the corresponding pseudocolor mapping of the temperature-dependent PL spectra. At low temperature, the lattice thermal vibration is weakened, the population number of phonons generated in the lattice is reduced, the electron–phonon coupling for carrier DOS becomes weak, and there are fewer defects or phonon scattering, resulting in a narrower FWHM and stronger intensity of the emission band. Therefore, the bi-mode emission characteristics are more obvious, and the emission intensity is higher at lower temperatures. As the temperature increases, the emission intensity decreases, and almost no bi-mode emission profile can be seen at 320 K, which is related to the thermal quenching of the emission state. It is worth noting that the 460 nm band also does not move, but the 600 nm band shifts from 600 to 620 nm at about 100–120 K, as shown in Figure 4b. This suggests that stronger electron–phonon coupling dominated for the phonon energy at about 110 K. This indicates the STE energy is temperature-dependent, and the stability of the emission color of the sample at different temperatures.

The Huang–Rhys factor (S) is a covariate describing the degree of Jahn−Teller lattice distortion and electron–phonon coupling in strong polar semiconductors [51]. To further reveal the influence of electron–phonon coupling on the optical properties of Cs_7_Cd_3_Br_13_:Sb^3+^/Pb^2+^, we calculated the relationship between FWHM and temperature using the following formula, and obtained the Huang−Rhys factor (S):

(4) $$\mathrm{FWHM}\;=\;2.36\sqrt{S}\hbar \omega_{\mathrm{phonon}\;}\sqrt{coth\left(\frac{\hbar \omega_{\mathrm{phonon}\;}}{2k_{B}T}\right)}$$

where *S* is the Huang–Rhys factor, which denotes the strength of the electron–phonon coupling, *ħω*_phonon_ is the average optical phonon energy, *k*_B_ is the Boltzmann constant, and *T* is the temperature. Figure 4c shows the FWHM versus temperature and the fitting results of the Sb^3+^ emission band. The fitting results of *S* is 11.6 and phonon frequency (*ℏω_phonon_*) is 56.3 meV (454.5 cm^−1^). The large phonon frequency (*ℏω_phonon_*) indicated the presence of large polaron formation with high-energy LO multiphonon involvement. The *S* values are close to the fitting values of most inorganic Sb^3+^ doped halides, indicating the presence of strong Jahn–Teller distortion of [SbBr_6_]^3−^ octahedron in the excited state, which leads to the wide-emission band and the large Stokes shift [36].

Figure 4d,e uses the temperature dependence on PL intensity at 490 and 620 nm and the reciprocal of temperature to fit *E*_b_ by the following formula [52]:

(5) $${\;\;I}_{\mathrm{PL}}\;=\;\frac{I_{0}}{1\;+\;A\exp\left(\;-\frac{E_{b}}{k_{B}T}\right)}$$

where *I*_PL_ is the PL spectral emission intensity at different temperatures, *I*_0_ is the PL spectral emission intensity at 0 K, *A* is the constant, *E*_b_ is the exciton binding energy, *k*_B_ is the Boltzmann constant, and *T* is the temperature. The fitting value of *E*_b (620)_ is 180 meV higher than that of *E*_b (490)_ (26.5 meV), which reflects that the doping state energy is higher at tetrahedra than at octahedra, indicating that the excitons of Sb^3+^ at octahedral sites are more resistant to heat than those of Pb^2+^.

Figure 4f shows the Raman spectra of Cs_7_Cd_3_Br_13_ and Cs_7_Cd_3_Br_13_:0.5%Sb^3+^, y%Pb^2+^ under 532 nm laser resonant excitation. It can be seen from Figure 4f that the lattice vibration mode of the doped sample does not change significantly after Sb^3+^ and Pb^2+^ replace Cd^2+^ [40]. Three Raman patterns are observed at 79, 150, and 184 cm^−1^. The 79 cm^−1^ pattern exhibits a blue shift after doping Sb^3+^. This may be due to the enhanced electron–phonon coupling leading to enhanced interaction between electrons and lattice vibration for small polaron formation, while the strongest in this range is the 184 cm^−1^ mode, which favors the formation of the large polaron for this phonon, coupled to the photoinduced carriers. Based on the temperature-dependent PL profile shown in Figure 4b, with the transition point at 100–120 K, we found that the small polaron by 79 cm^−1^ mode is critical for this system, but not the 184 cm^−1^ mode. This implies that the latter mode shows a minor ratio for the polaron formation. The 79 cm^−1^ mode is far from the value of 56.3 meV fitted from Equation (3); this difference indicates multiphonon coupling effect dominates in the STE emission, especially in the Sb^3+^ sites, which represents a significant nonlinear effect in their electron–phonon coupling and other physical interactions, just as in those perovskite structures [53,54].

**Figure 5:**
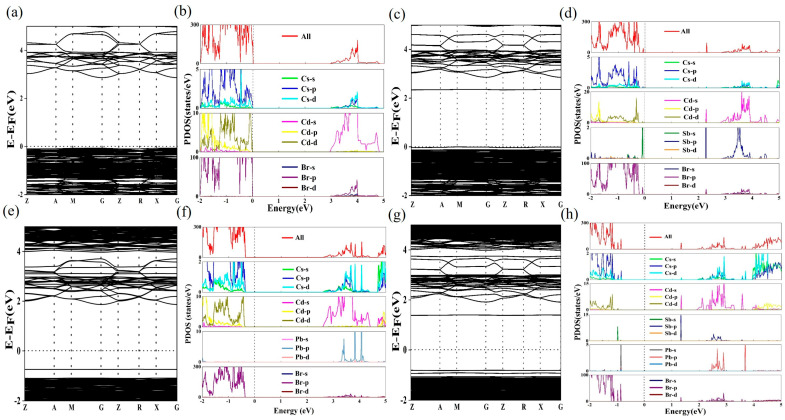
Band structures of (**a**) Cs_7_Cd_3_Br_13_, (**c**) Sb^3+^ doped Cs_7_Cd_3_Br_13_, (**e**) Pb^2+^ doped Cs_7_Cd_3_Br_13_, (**g**) Sb^3+^/Pb^2+^ co-doped Cs_7_Cd_3_Br_13_. Total density of states (TDOS) and projected density of states (PDOS) of (**b**) Cs_7_Cd_3_Br_13_, (**d**) Sb^3+^ doped Cs_7_Cd_3_Br_13_, (**f**) Pb^2+^ doped Cs_7_Cd_3_Br_13_, (**h**) Sb^3+^/Pb^2+^ co-doped Cs_7_Cd_3_Br_13_.

To elucidate and compare the optical properties and photoluminescence mechanism of Cs_7_Cd_3_Br_13_, Cs_7_Cd_3_Br_13_:Sb^3+^, Cs_7_Cd_3_Br_13_:Pb^2+^, and Cs_7_Cd_3_Br_13_:Sb^3+^/Pb^2+^, we simulated the doped samples by replacing Cd atoms with Sb atoms and Pb atoms in the lattice, and calculated their electron band structures and corresponding projected state density (PDOS) of these complexes using DFT, as shown in Figure 5. The band gap of Cs_7_Cd_3_Br_13_ is calculated to be 2.93 eV (Figure 5a), which is lower than the band gap estimated experimentally because standard DFT calculations often underestimate the band gap of the material. The PDOS results in Figure 5b show that the valence band maximum (VBM) of Cs_7_Cd_3_Br_13_ is mainly composed of Br-p orbital, and the conduction band minimum (CBM) is mainly composed of Cd-s orbital [43]. After the addition of Sb^3+^, the band gap value decreases to 2.35 eV (Figure 5c). Compared with undoped Cs_7_Cd_3_Br_13_, a new impurity band appears in the conduction band of Sb^3+^ after doping to form a new conduction band base, which is mainly composed of Cd-s, Sb-p, and Cl-p orbitals. The appearance of this new impurity band changes the transition properties of the compound and increases the probability of radiation recombination. These indicate that the band edges of CBM and VBM are both composed of the Cd states, though the Sb 5s^2^ is located at the edge of the VBM. The radiative transition of the excited Cd state to the Sb 5s^2^ state may be low for their indirect nature and low Sb concentration, while the minor Sb excited state to Sb 5s^2^ state transition is allowed. That is the cause of its low emission efficiency of this material. When Pb^2+^ is doped into the system, the band gap is 2.59 eV, with the VBM contributed by the Cd-d and Br-p orbitals at octahedra, and the CBM contributed by the Cd-d and Pb-s orbitals at tetrahedra. Therefore, in the Sb^3+^/Pb^2+^ co-doped Cs_7_Cd_3_Br_13_ system, the VBM is contributed by Cd-d, Pb-s, and Br-p orbitals [55], whereas the CBM is contributed by Cd-s, Sb-p, but no Pb-p orbitals, and hence though the band gap is reduced to 1.34 eV, but gives not highly efficient emission at Pb sites because the lowest level of the conduction band is not from the Pb sites. In the PLE spectra for different emission bands, their individual energy levels are distributed sparsely in the range of 300–520 nm, which is why they can give emission with different colors at different excitations. This PLE profile is in agreement with their DFT calculated band structures as shown in Figure 5. Only Cs ions in the lattice play important roles in confining the doped states in the octahedra and tetrahedra for emission, but not by the matrix of Cd-Cl clusters in this Sb and Pb doped Cd halide.

**Figure 6:**
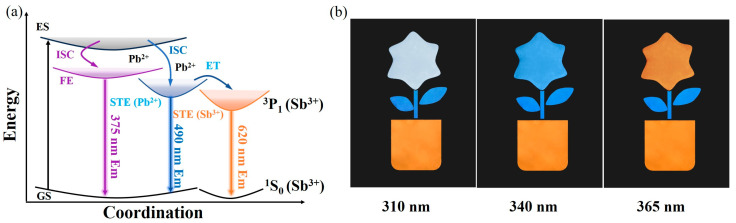
(**a**) Diagram of the luminescence mechanism of Cs_7_Cd_3_Br_13_:Sb^3+^/Pb^2+^. (**b**) Fluorescent anti-counterfeiting patterns (“potted”) made by Cs_7_Cd_3_Br_13_:0.5%Sb^3+^ (pot), Cs_7_Cd_3_Br_13_:5%Pb^2+^ (leaves), and Cs_7_Cd_3_Br_13_:0.5%Sb^3+^, 4%Pb^2+^ (flowers).

Figure 6a shows the luminescence mechanism of Cs_7_Cd_3_Br_13_:Sb^3+^/Pb^2+^. Under UV light excitation, electrons are pumped from the valence band to the conduction band and then transferred to the STE via nonradiative relaxation, resulting in the emission of the intrinsic weak STE1 emission of the host Cs_7_Cd_3_Br_13_ at Cd site, FE and STE2 emission by the Jahn–Teller effect caused by the Pb^2+^ incorporation is not high because the lowest conduction band minimum is composed mainly Cd but not Pb, though bandgap is reduced. Furthermore, optical characterization reveals that FEs and STE2 of Pb^2+^ can be transferred to STE3 of Sb^3+^ through energy transfer to lower levels of Sb sites, resulting in triplet state emission of Sb^3+^ (^3^P_1_→^1^S_0_ transition) because the Sb 5s^2^ state is at the VBM. Thus, single-component powders of Cs_7_Cd_3_Br_13_:Sb^3+^, Pb^2+^ with multiple PL emission centers can achieve white light when both doped metal ions working as the emission centers, i.e., FE and STE2 emission induced by Pb^2+^, STE3 emission from the triplet state (^3^P_1_→^1^S_0_) induced by Sb^3+^, and slightly orange STE1 emission from the undoped Cs_7_Cd_3_Br_13_, but its emission efficiency is not high because the lowest levels are Cd ions in the octahedra sites. In addition, tunable emission is observed in Cs_7_Cd_3_Br_13_:Sb^3+^, Pb^2+^ samples due to their individual excitons confined by Cs ions, and their energy transfer from STE2 to STE3 for emission of Sb^3+^.

The XRD spectra and PL spectra of the three samples after being stored at room temperature for one month are shown in Figure S13. The results indicate that the PL intensity has only slightly decreased, and the XRD spectra do not show any changes, which is consistent with the initial synthesis. These results suggest that these three samples have good structural and luminescence stability at room temperature. In this work, tunable multimode emission was successfully achieved by adjusting the Sb^3+^/Pb^2+^ co-doping ratio in Cs_7_Cd_3_Br_13_. The sample of Cs_7_Cd_3_Br_13_:0.5%Sb^3+^ exhibited only a single orange light emission from Sb sites at different excitation wavelengths of 310, 340, and 365 nm. In addition, the sample of Cs_7_Cd_3_Br_13_:5%Pb^2+^ only shows blue emission at the above three different excitation wavelengths. However, the sample of Cs_7_Cd_3_Br_13_:0.5%Sb^3+^, 4%Pb^2+^ exhibits white, blue, and orange emission at these three excitation wavelengths, respectively. Using these unique luminescent color conversion properties, we used a hollow template with a “potted” pattern to demonstrate the effect of the fluorescent anti-counterfeiting. The three fluorescent powders of Cs_7_Cd_3_Br_13_:0.5%Sb^3+^, Cs_7_Cd_3_Br_13_:5%Pb^2+^, and Cs_7_Cd_3_Br_13_:0.5%Sb^3+^,4%Pb^2+^ were combined to make a “potted” pattern, as shown in Figure 6b. The mixture of Cs_7_Cd_3_Br_13_:0.5%Sb^3+^, 4%Pb^2+^ powder and epoxy resin was filled into the “flower” region, while the mixture of Cs_7_Cd_3_Br_13_:5%Pb^2+^ powder and epoxy resin was filled into the “leaf” region. For the “pot” region, a mixture of Cs_7_Cd_3_Br_13_:0.5%Sb^3+^ powder and epoxy resin was used for filling. Subsequently, the hollow template and the black cardboard base were placed inside a lightbox in a darkroom. The lightbox equipped with UV light sources at 310, 340, and 365 nm was used. As shown in the photograph in Figure 6b, under UV excitation at 310, 340, and 365 nm, only the “flower” changed from white and blue to orange, while the “leaf” and “pot” remained blue and orange, respectively. Therefore, based on Cs_7_Cd_3_Br_13_:Sb^3+^/Pb^2+^, three-level anti-counterfeiting can be achieved, which can ensure the safety of the product.

## 4. Conclusions

In summary, we successfully synthesized Sb^3+^/Pb^2+^ co-doped Cs_7_Cd_3_Br_13_ perovskite powder using a simple hydrothermal method. This powder exhibits unique luminescent properties. After doping with Sb^3+^ ions, the Cs_7_Cd_3_Br_13_ powder displays a strong orange wide-emission band at 620 nm. When introducing different concentrations of Pb^2+^ ions, the excitons of both Sb^3+^ and Pb^2+^ are confined by Cs ions, and energy transfer from STE2 to STE3. This results in tunable emission colors from orange to white and then to blue. Notably, the optimized Cs_7_Cd_3_Br_13_:0.5% Sb^3+^, 4% Pb^2+^ powder shows significant excitation-wavelength dependence, enabling tunable emission within a single component. Based on this unique luminescent property, we demonstrated its potential application in anti-counterfeiting. Additionally, because of the lowest energy levels of Cd ions in the chained octahedra, these ions exhibit less efficient emission, leading to a maximum PLQY of 17.2%. Therefore, this study not only broadens the understanding of Cd-based halides but also provides a new pathway for achieving tunable emission through the co-doping strategy of metal ions.

## Data Availability

The data supporting this article have been included as part of the Supplementary Materials.

## Supplementary Materials

The following supporting information can be downloaded at: https://www.mdpi.com/article/10.3390/nano15161238/s1. Figure S1. XRD spectra of Cs_7_Cd_3_Br_13_:0.5%Sb^3+^, y%Pb^2+^ (y = 2.5, 3, 4, 5, and 5.5). Figure S2. (a) SEM image and component elemental maps of a Cs_7_Cd_3_Br_13_ single particle. (b) Energy dispersive spectroscopy of Cs_7_Cd_3_Br_13_. Figure S3. Energy dispersive spectroscopy of Cs_7_Cd_3_Br_13_:0.5%Sb^3+^, 4%Pb^2+^. Figure S4. (a–f) The high-resolution XPS spectra of Cs 3d, Cd 3d, Br 3d, Sb 3d and Pb 4f of Cs_7_Cd_3_Br_13_ (black) and Cs_7_Cd_3_Br_13_:0.5%Sb^3+^, 4%Pb^2+^ (red). Figure S5. (a) PL lifetime decay curves for different concentrations of Sb^3+^ doped Cs_7_Cd_3_Br_13_ at room temperature (λ_ex_ = 370 nm, λ_em_ = 620 nm). (b) PL lifetime decay curves for different concentration Pb^2+^ doped Cs_7_Cd_3_Br_13_ at room temperature (λ_ex_ = 370 nm, λ_em_ = 460 nm). Table S1. Cs_7_Cd_3_Br_13_:x%Sb^3+^ (x = 0, 0.3, 0.5, 0.8, and 1) PL lifetime decay fitting results (λ_ex_ = 370 nm, λ_em_ = 620 nm). Table S2. Cs_7_Cd_3_Br_13_:y%Pb^2+^ (y = 2, 3, 4, 5, and 6) PL lifetime decay fitting results (λ_ex_ = 340 nm, λ_em_ = 460 nm). Figure S6. The normalized PL spectra of Cs_7_Cd_3_Br_13_:0.5%Sb^3+^, y%Pb^2+^ (y = 2.5, 3, 4, 5, and 5.5) at 310 nm excitation. Figure S7. (a) CIE coordinates of Cs_7_Cd_3_Br_13_:0.5%Sb^3+^, y%Pb^2+^ (y = 2.5, 3, 4, 5 and 5.5). (b) CIE coordinates of Cs_7_Cd_3_Br_13_:0.5%Sb^3+^, 4%Pb^2+^. Figure S8. The concentration-dependent emission spectra of Cs_7_Cd_3_Br_13_:0.5%Sb^3+^, y%Pb^2+^ (y = 2.5, 3, 4, 5, and 5.5) (a) at 340 nm and (b) 370 nm excitation. Figure S9. The concentration-dependent PLE spectra of Cs_7_Cd_3_Br_13_:0.5%Sb^3+^, y%Pb^2+^ (y = 2.5, 3, 4, 5, and 5.5) under (a) 490 nm and (b) 620 nm emission. Figure S10. The normalized PLE spectra of Cs_7_Cd_3_Br_13_:0.5%Sb^3+^, y%Pb^2+^ (y = 2.5, 3, 4, 5, and 5.5) under (a) 490 nm and (b) 620 nm emission. Figure S11. PLQY of Cs_7_Cd_3_Br_13_:0.5%Sb^3+^, y%Pb^2+^ single crystals versus Pb^2+^ content. Table S3. Cs_7_Cd_3_Br_13_:0.5%Sb^3+^, y%Pb^2+^ (y = 2.5, 3, 4, 5, and 5.5) PL lifetime decay fitting results (λ_ex_ = 310 nm, λ_em_ = 490 nm). Table S4. Cs_7_Cd_3_Br_13_:0.5%Sb^3+^, y%Pb^2+^ (y = 2.5, 3, 4, 5, and 5.5) PL lifetime decay fitting results (λ_ex_ = 310 nm, λ_em_ = 620 nm). Figure S12. (a) Temperature-dependent PL spectra of Cs_7_Cd_3_Br_13_ (T = 80–320 K, λ_ex_ = 370 nm). (b) Temperature-dependent PL spectra of Cs_7_Cd_3_Br_13_:5% Pb^2+^ (T = 80–340 K, λ_ex_ = 340 nm). Figure S13. (a–c) XRD spectra of Cs_7_Cd_3_Br_13_:0.5%Sb^3+^, 4%Pb^2+^, Cs_7_Cd_3_Br_13_:0.5%Sb^3+^, and Cs_7_Cd_3_Br_13_:5%Pb^2+^ before and after one month at room temperature; (d–f) PL spectra of Cs_7_Cd_3_Br_13_:0.5%Sb^3+^, 4%Pb^2+^, Cs_7_Cd_3_Br_13_:0.5%Sb^3+^, and Cs_7_Cd_3_Br_13_:5%Pb^2+^ before and after one month at room temperature.
