# Supplementary material for: Tunable Emission Properties of Sb3+/Pb2+ Co-Doped Cs7Cd3Br13 for Optical Anti-Counterfeiting Application
Source: Nanomaterials (Basel). 2025 Aug 13;15(16):1238. doi: 10.3390/nano15161238 (PMC12388824; doi:10.3390/nano15161238)
Supplement: Supplementary file 1 [file nanomaterials-15-01238-s001.zip › nanomaterials-3766858-supplementary.pdf]

Supplementary Material

Tunable emission properties of Sb^{3+}/Pb^{2+} co-doped $Cs_7Cd_3Br_{13}$ for optical anti-counterfeiting application

Bingbing Zheng [1], Shuaigang Ge [1], Lingli Chen [1], Yijia Wen [1], Kaihuang Huang [1] and Bingsuo Zou [1,2,*]

[1] School of Physical Science and Technology, Guangxi University, Nanning 530004, China

[2] State Key Laboratory of Featured Metal Materials and Life-cycle Safety for Composite Structures, MOE Key Laboratory of New Processing Technology for Nonferrous Metals and Materials, and School of Resources, Environment and Materials, Guangxi University, Nanning 530004, China

Measurement and Characterization.

The powder X-ray diffraction (PXRD) pattern of $Cs_7Cd_3Br_{13}$: x%Sb^{3+}, y%Pb^{2+} were obtained by using a SMARTLAB 3KW X-ray diffractometer with Cu Kα radiation (λ=1.54059 Å) and scanning the 2θ range of 5-70° at 10°/min. The morphologies and elemental content of the samples were characterized by a Hitachi SU8020 field emission scanning electron microscope (SEM) and an Oxford X-Max Aztec energy dispersive X-ray spectroscopy (EDS) at a voltage of 10 kV and a current of 10 mA respectively. The Thermo-Fisher Scientific ESCALAB 250Xi Xray photoelectron spectroscopy (XPS) was used to determine the element type, element content and chemical bond state on the surface of the compound. The PL emission spectrum, temperature-dependent PL spectra, PL excitation (PLE) spectra, PL lifetime spectra and PLQYs were achieved via the Horiba Jobin Yvon Fluorolog-3 spectrometer and Edinburgh FLS-1000 spectrofluorometer. The absorption spectra were measured by the Lambda 750 ultraviolet-visible spectrophotometer with a range from 220 nm to 800 nm. The Raman spectroscopy and excitation power-dependent PL spectra were performed on the WITec alpha300R Raman fluorescence spectrometer by using an excitation wavelength of 532 nm.

Computational Methods.

All calculations at density functional theory are carried out using the Vienna Ab initio simulation package (VASP). The generalized gradient approximation of the Perdew-Burke-Ernzerhof (PBE) parameterization with projector-augmented wave method are performed for the exchange and correlation functional. The ultra-soft pseudopotential

is used for Cs, Cd, Sb, Pb and Br elements. For $Cs_7Cd_3Br_{13}$, $Cs_7Cd_3Br_{13}$:0.5%Sb^{3+} and $Cs_7Cd_3Br_{13}$:4%Pb^{2+} and $Cs_7Cd_3Br_{13}$:0.5%Sb^{3+}, 4%Pb^{2+}, a 1 × 1 × 2 supercell with 184 atoms were constructed. The kinetic-energy cutoff of 400 eV and a 3×3×2 Monkhorst-Pack k-mesh for the wavefunction basis set is employed. The energy convergence criterion is set as 1.0×10^{-5} eV for structural relaxations.

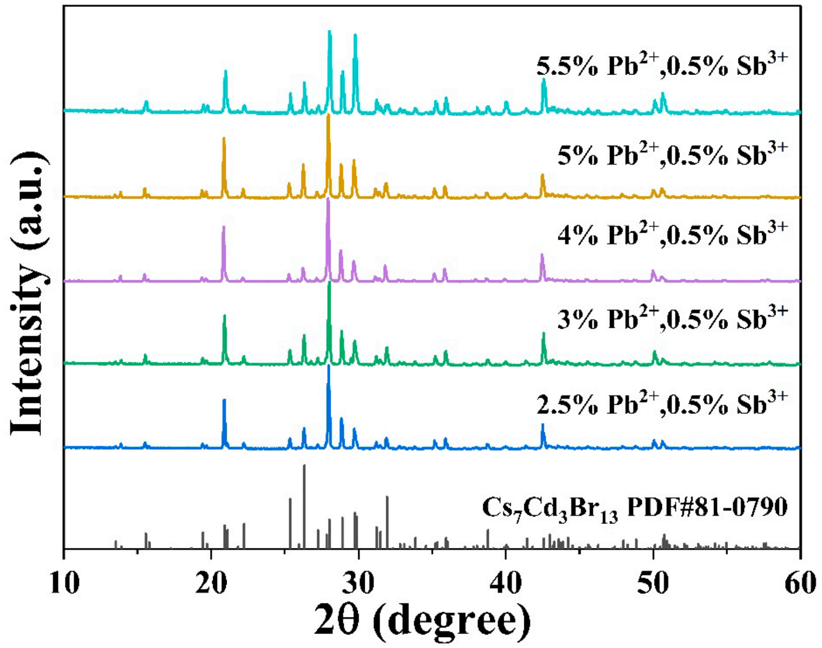

Figure S1. XRD spectra of $Cs_7Cd_3Br_{13}$:0.5%Sb^{3+}, y%Pb^{2+} (y=2.5, 3, 4, 5 and 5.5).

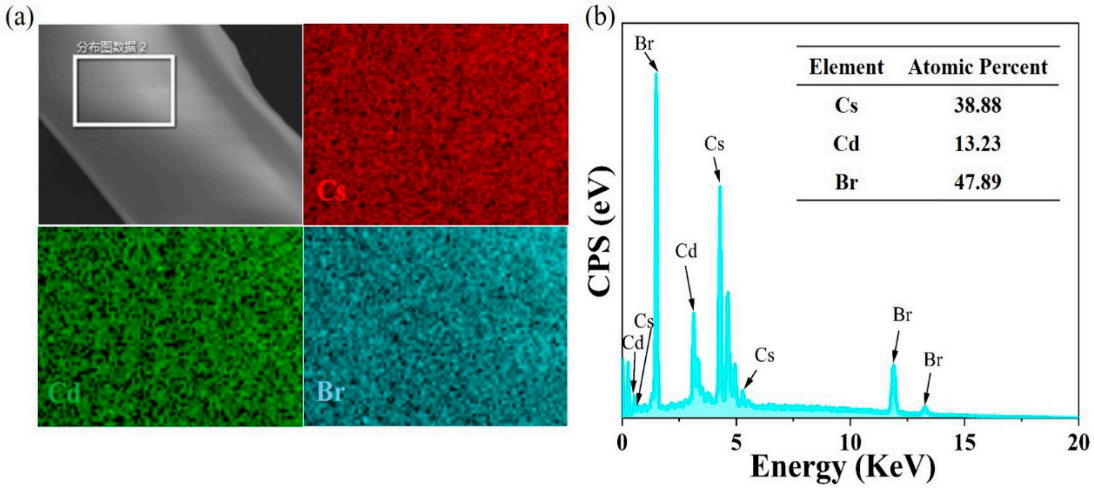

Figure S2. (a) SEM image and component elemental maps of a $Cs_7Cd_3Br_{13}$ single particle. (b) Energy dispersive spectroscopy of $Cs_7Cd_3Br_{13}$.

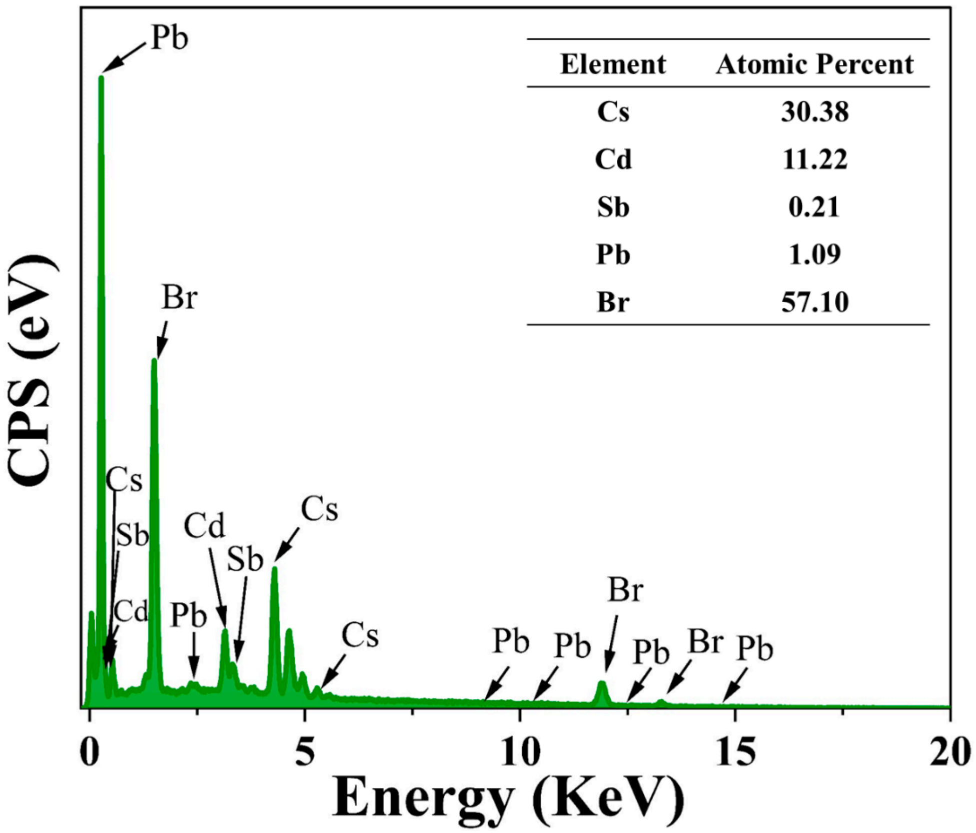

Figure S3. Energy dispersive spectroscopy of $Cs_7Cd_3Br_{13}$:0.5%Sb^{3+}, 4%Pb^{2+}.

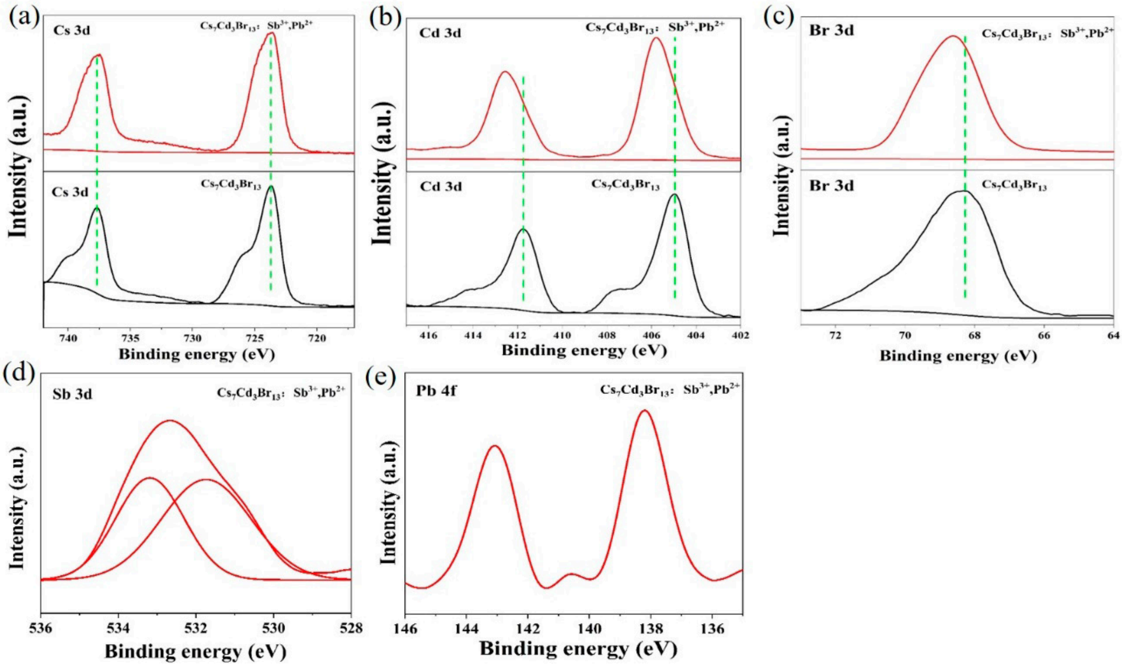

Figure S4. (a-f) The high-resolution XPS spectra of Cs 3d, Cd 3d, Br 3d, Sb 3d and Pb 4f of $Cs_7Cd_3Br_{13}$ (black) and $Cs_7Cd_3Br_{13}$:0.5%Sb^{3+}, 4%Pb^{2+} (red).

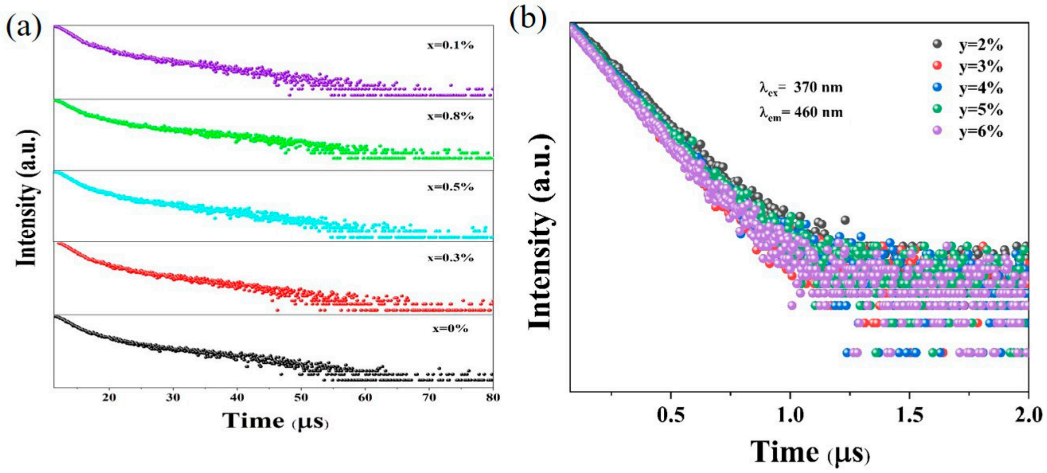

Figure S5. (a) PL lifetime decay curves for different concentration Sb^{3+} doped $Cs_7Cd_3Br_{13}$ at room temperature (λ_{ex}=370 nm, λ_{em}=620 nm). (b) PL lifetime decay curves for different concentration Pb^{2+} doped $Cs_7Cd_3Br_{13}$ at room temperature (λ_{ex} =370 nm, λ_{em}=460 nm).

Table S1. $Cs_7Cd_3Br_{13}$:x%Sb^{3+} (x=0, 0.3, 0.5, 0.8 and 1) PL lifetime decay fitting results (λ_{ex}=370 nm, λ_{em}=620 nm).

X (%)	A_1 (%)	τ_1 (μs)	A_2 (%)	τ_2 (μs)	τ_{ave} (μs)
0		2.80			
0.3	0.13	15.21	99.87	2.46	2.57
0.5	99.89	2.58	0.11	18.72	2.70
0.8	0.10	15.38	99.90	2.37	2.46
1	0.10	19.41	99.90	2.56	2.67

Table S2. $Cs_7Cd_3Br_{13}$:y%Pb^{2+} (y=2, 3, 4, 5 and 6) PL lifetime decay fitting results (λ_{ex}=340 nm, λ_{em}=460 nm).

Y(%)	A_1 (%)	τ_1 (ns)	A_2 (%)	τ_2 (ns)	τ_{ave} (ns)
2	50.61	180.27	49.39	180.27	180.27
3	54.32	155.81	45.68	155.81	155.81
4	66.86	162.38	33.14	162.39	162.38
5	39.62	165.12	60.38	165.12	165.12
6	51.26	155.03	48.74	155.03	155.03

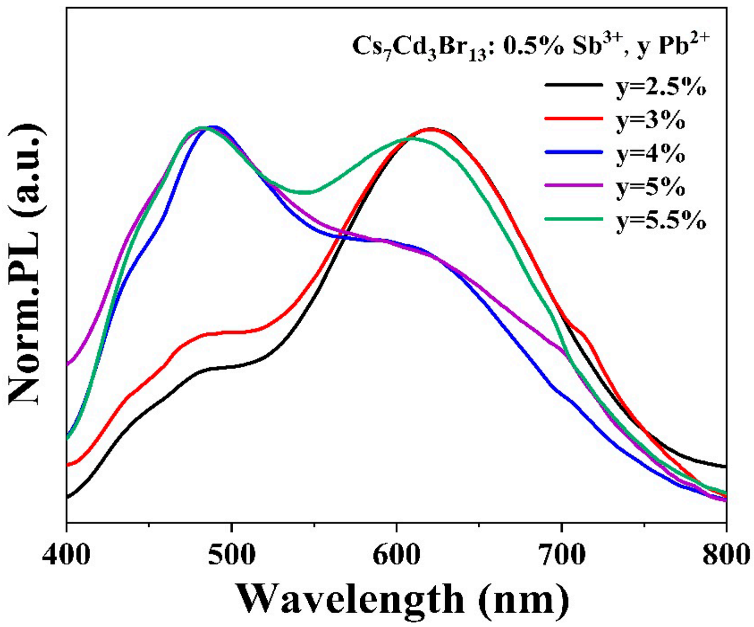

Figure S6. The normalized PL spectra of $Cs_7Cd_3Br_{13}$:0.5%Sb^{3+}, y%Pb^{2+} (y=2.5, 3, 4, 5 and 5.5) at 310 nm excitation.

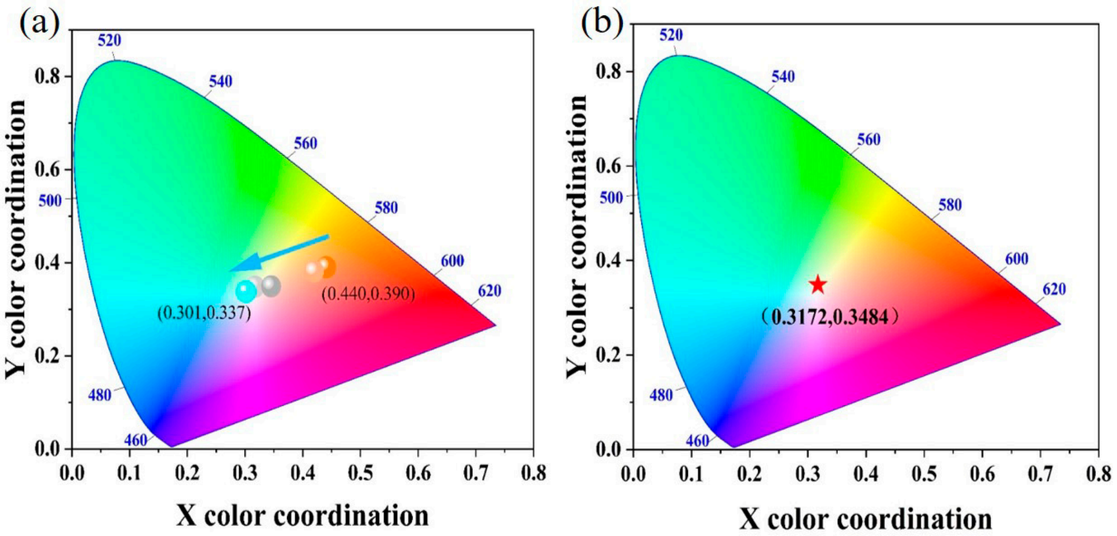

Figure S7. (a) CIE coordinates of $Cs_7Cd_3Br_{13}$:0.5%Sb^{3+}, y%Pb^{2+} (y=2.5, 3, 4, 5 and 5.5). (b) CIE coordinates of $Cs_7Cd_3Br_{13}$:0.5%Sb^{3+}, 4%Pb^{2+}.

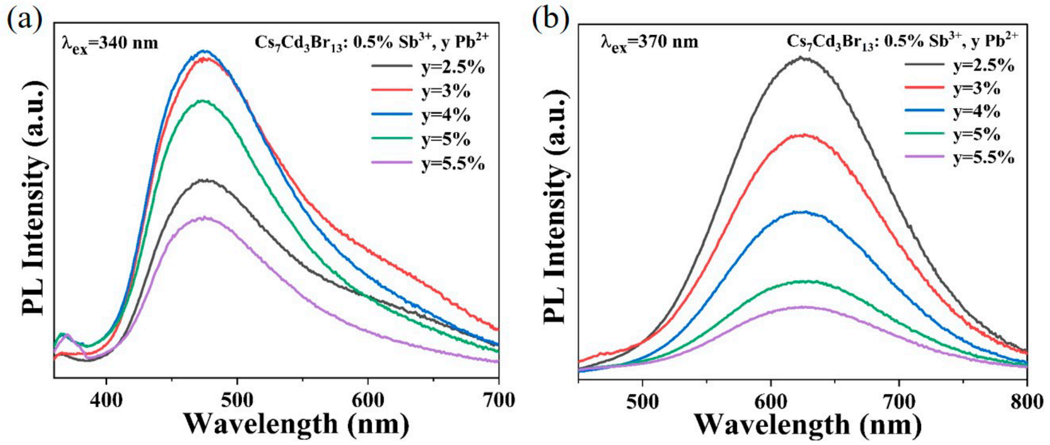

Figure S8. The concentration-dependent PL spectra of $Cs_7Cd_3Br_{13}$:0.5%Sb^{3+}, y%Pb^{2+} (y=2.5, 3, 4, 5 and 5.5) (a) at 340 nm and (b) 370 nm excitation.

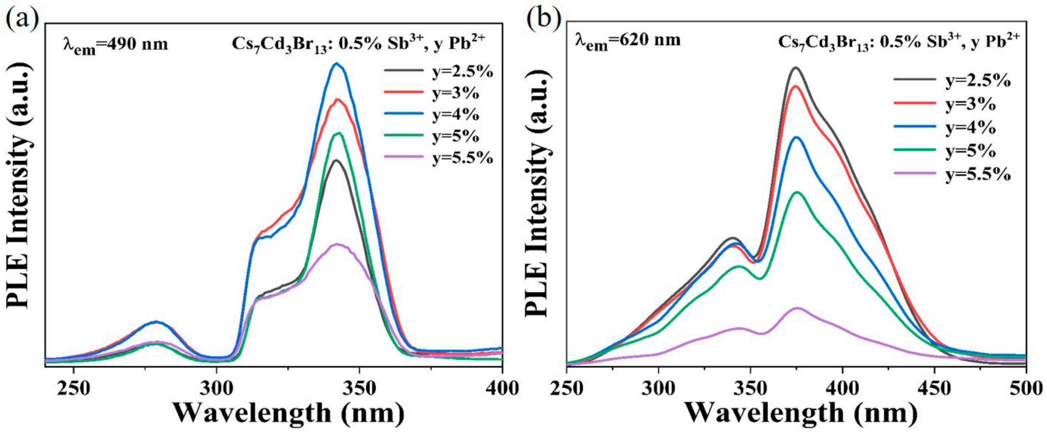

Figure S9. The concentration-dependent PLE spectra of $Cs_7Cd_3Br_{13}$:0.5%Sb^{3+}, y%Pb^{2+} (y=2.5, 3, 4, 5 and 5.5) under (a) 490 nm and (b) 620 nm emission.

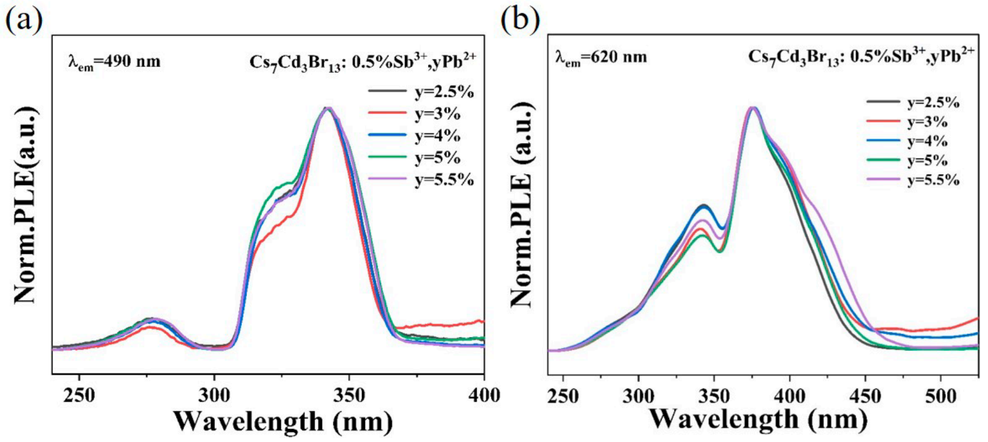

Figure S10. The normalized PLE spectra of $Cs_7Cd_3Br_{13}$:0.5%Sb^{3+}, y%Pb^{2+} (y=2.5, 3, 4, 5 and 5.5) under (a) 490 nm and (b) 620 nm emission.

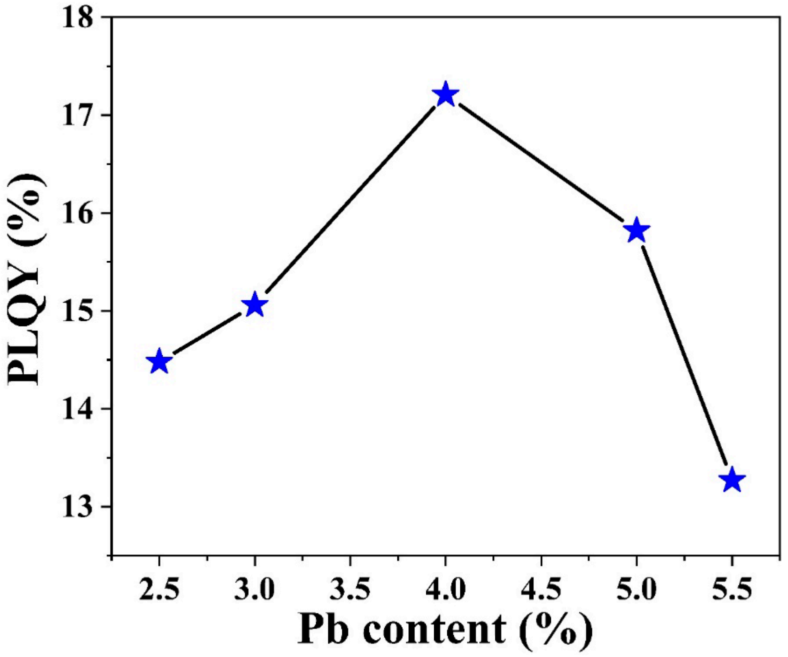

Figure S11. PLQY of $Cs_7Cd_3Br_{13}$:0.5%Sb^{3+}, y%Pb^{2+} single crystals versus Pb^{2+} content.

Table S3. $Cs_7Cd_3Br_{13}$:0.5%Sb^{3+}, y%Pb^{2+} (y=2.5, 3, 4, 5 and 5.5) PL lifetime decay fitting results (λ_{ex}=310 nm, λ_{em}=490 nm).

Y(%)	A_1 (%)	τ_1 (ns)	A_2 (%)	τ_2 (ns)	τ_{ave} (ns)
2.5	72.72	56.43	27.28	124.05	86.98
3	87.36	97.54	12.64	192.61	118.65
4	65.88	81.10	34.12	160.11	121.04
5	75.67	68.41	24.33	143.94	98.88
5.5	66.96	61.16	33.04	129.66	96.18

Table S4. $Cs_7Cd_3Br_{13}$:0.5%Sb^{3+}, y%Pb^{2+} (y=2.5, 3, 4, 5 and 5.5) PL lifetime decay fitting results (λ_{ex}=310 nm, λ_{em}=620 nm).

Y(%)	A_1 (%)	τ_1 (ns)	A_2 (%)	τ_2 (ns)	τ_{ave} (ns)
2.5	80.33	158.11	19.67	895.83	586.74
3	81.72	138.67	18.28	822.30	528.38
4	84.68	118.42	15.32	780.17	478.20
5	87.55	109.48	12.45	689.30	383.35
5.5	88.88	96.87	11.12	653.9	351.78

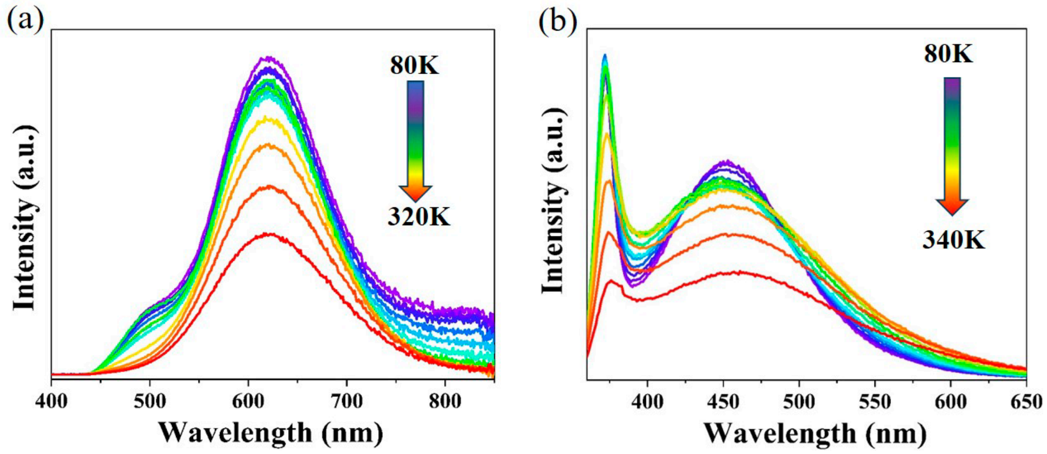

Figure S12. (a) Temperature-dependent PL spectra of $Cs_7Cd_3Br_{13}$ (T=80-320 K, λ_{ex}=370 nm). (b) Temperature-dependent PL spectra of $Cs_7Cd_3Br_{13}$:5% Pb^{2+} (T=80-340 K, λ_{ex}=340 nm).

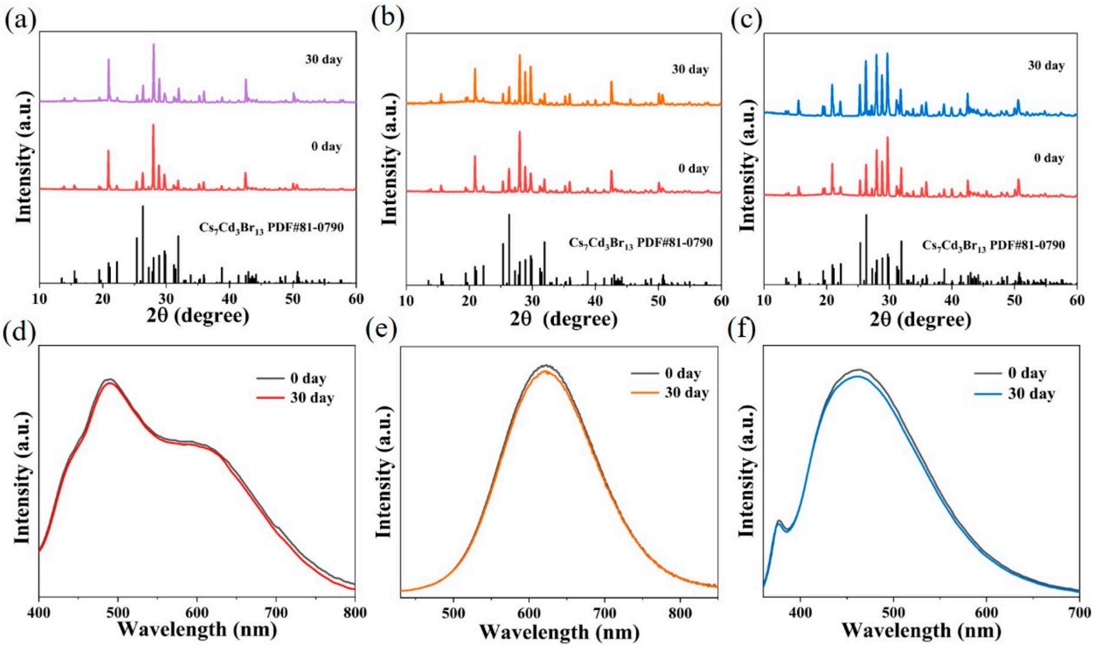

Figure S13. (a-c) XRD spectra of $Cs_7Cd_3Br_{13}$:0.5%Sb^{3+}, 4%Pb^{2+}, $Cs_7Cd_3Br_{13}$:0.5%Sb^{3+}, and $Cs_7Cd_3Br_{13}$:5%Pb^{2+} before and after one month at room temperature; (d-f) PL spectra of $Cs_7Cd_3Br_{13}$:0.5%Sb^{3+}, 4%Pb^{2+}, $Cs_7Cd_3Br_{13}$:0.5%Sb^{3+}, and $Cs_7Cd_3Br_{13}$:5%Pb^{2+} before and after one month at room temperature.
